# CaMKII inhibition with KN93 attenuates endothelin and serotonin receptor-mediated vasoconstriction and prevents subarachnoid hemorrhage-induced deficits in sensorimotor function

**DOI:** 10.1186/s12974-014-0207-2

**Published:** 2014-12-10

**Authors:** Lars Edvinsson, Gro Klitgaard Povlsen, Hilda Ahnstedt, Roya Waldsee

**Affiliations:** Division of Experimental Vascular Research, Department of Clinical Sciences, Lund University and Lund University Hospital, SE-221 84 Lund, Sweden; Department of Clinical Experimental Research, Glostrup University Hospital, Nordre Ringvej 69, 2600 Glostrup, Denmark

**Keywords:** CaMKII, Delayed cerebral ischemia, DREAM, Endothelin receptors, KN93, Serotonin receptors, Subarachnoid hemorrhage, Vasospasm

## Abstract

**Background:**

It has been suggested that transcriptional upregulation of cerebral artery contractile endothelin (ET_B_) and 5-hydroxytryptamine (5-HT_1B_) receptors play an important role in the development of late cerebral ischemia and increased vasoconstriction after subarachnoid hemorrhage (SAH). We tested the hypothesis that inhibition of calcium calmodulin-dependent protein kinase II (CaMKII) may reduce cerebral vasoconstriction mediated by endothelin and serotonin receptors and improve neurological outcome after experimental SAH.

**Methods:**

SAH was induced in adult rats by injection of 250 μL autologous blood into the basal cisterns. The CaMKII activity in cerebral vessels was studied by Western blot and immunohistochemistry. The vasomotor responses of middle cerebral and basilar arteries were measured in a sensitive myograph system. The functional outcome was examined by the rotating pole test 2 and 3 days after SAH.

**Results:**

SAH induced a rapid early increase in phosphorylated CaMKII protein at 1 h that was attenuated by cisternal administration of the CaMKII inhibitor KN93 (0.501 μg/kg) 45 min prior and immediately after SAH as evaluated by Western blot. Application of KN93 at 1 h and every 12 h post-SAH significantly reduced vascular CaMKII immunoreactivity at 72 h. In addition, contractile responses of cerebral arteries to endothelin-1 (ET-1) and 5-hydroxycarboxamide (5-CT) were increased at this time-point. KN93 treatment significantly attenuated the contraction induced by ET-1 and 5-CT. Importantly, treatment with the CaMKII inhibitor prevented SAH-induced deficits in neurological function, as evaluated by the rotating pole test, and similar sensorimotor scores were seen in sham-operated animals.

**Conclusions:**

The present study has shown that SAH is associated with increased contractile responses to ET-1 and 5-CT in cerebral arteries and enhanced early activation of CaMKII. Treatment with the CaMKII inhibitor KN93 attenuated the contractile responses and prevented impaired sensorimotor function after SAH.

## Background

Subarachnoid hemorrhage (SAH) is associated with high initial mortality (15%) and after several days the development of delayed cerebral ischemia (DCI) and cerebral vasospasm (CVS) causes further considerable morbidity and mortality [1]. The pathophysiological mechanisms responsible for DCI and CVS are still elusive, although a number of associated factors have been revealed such as enhanced levels of ions or free radicals [2,3], upregulation of contractile endothelin receptors [4], reduced levels of endothelial relaxant factors [5], elevation of circulatory and/or local messenger molecules such as endothelin-1 (ET-1) and 5-hydroxytryptamine (5-HT) [6], and imbalance between vasoconstrictors and vasodilators [1]. Hence, the mechanisms behind DCI after SAH are likely complex and multifactorial [7].

ET-1 and 5-HT are potent cerebral vasoconstrictors which mediate strong vasomotor responses through their respective receptors; endothelin A (ET_A_), endothelin B (ET_B_), 5-HT_1B_, and 5-HT_2A_ receptors in rat cerebral arteries [8]. It has been demonstrated that experimental SAH induces upregulation of contractile ET_B_ and 5-HT_1B_ receptors in the smooth muscle cells of cerebral arteries [9]. The phenomenon of enhanced receptor expression is thus not limited to only some receptor subtypes but also associated with cerebrovascular inflammation [10]. Thus, the responses to SAH are complex and, therefore, studies designed to test a single specific target of vasoconstrictor receptor subtypes have been unsuccessful in improving outcome after SAH [11]. Most recently, the CONSCIOUSS trials using the ET_A_ receptor antagonist clazosentan failed to improve clinical outcome after SAH and thereby brought an end to the long-standing hope of helping SAH patients by direct targeting of endothelin receptors [12]. Novel approaches to the treatment of DCI are therefore urgently needed.

The MEK/ERK1/2 protein kinase pathway has been observed to play a central role in the upregulation of cerebrovascular vasoconstrictor receptors and inflammation pathways and is associated with DCI after SAH [13]. We have found that cerebral arteries undergo vascular phenotypic changes after SAH and ischemic stroke [7]. In a previous study, we have developed a method, using organ culture, by which cerebrovascular receptor upregulation can be examined *in vitro* [14-17]. By using this *in vitro* model, it was observed that calcium calmodulin-dependent protein kinase (CaMK) II is associated with upregulation of contractile ET_B_ receptors, activation of inflammation pathways, and the TNFα receptor 1 [10]. In addition, we reported that the CaMKII and MEK1/2 pathways may interact or cross-talk in this process [10].

CaMKII is a multifunctional serine/threonine kinase with various cellular functions, including gene expression, cell cycle control, and hormone production [18]. It has been shown that CaMKII has a significant effect on downstream regulatory element antagonist modulator (DREAM), which is a Ca^2+^-regulated transcription repressor. Interestingly, it has been shown that CaMKII inhibitors reduce upregulation of contractile ET_B_ receptors [19] and has a neuroprotective effect after experimental ischemic stroke [20]. CaMKII is important for regulation of intracellular Ca^2+^ homeostasis in the overall regulation of vascular smooth muscle cell contraction [21]; however, we postulate that, in addition, CaMKII may participate in expression of vascular endothelin receptors and cerebrovascular inflammation responses.

The aim of the present study was to address the hypothesis that inhibition of CaMKII with KN93 *in vivo* prevents CaMKII activation and reduces DREAM expression in cerebral arteries after SAH, reduces vasoconstriction mediated by endothelin and serotonin receptors, and improves functional outcome after SAH.

## Materials and methods

### Rat SAH model

All procedures were performed strictly within national laws and guidelines and were approved by the Danish Animal Experimentation Inspectorate (license no. 2011/561-2025).

SAH was induced as previously described [22]. Briefly, male Sprague-Dawley rats, weighing 320 to 340 g body weight, were anesthetized using 4% isoflurane (Abbott Laboratories, Illinois USA) in atmospheric air/O_2_ (70:30). The rats were orally intubated and kept on artificial ventilation with inhalation of 1 to 2% isoflurane in N_2_O/O_2_ (70:30) during the surgery. Respiration was monitored by regular withdrawing of blood samples to a blood gas analyzer (Radiometer, Copenhagen, Denmark). One catheter was placed in the tail artery to measure blood pressure and another in the cisterna magna to measure the intracranial pressure (ICP). On the right side of the skull, 4 mm anterior from bregma and 3 mm to the midline, a hole was drilled through the bone to the dura mater without perforation and a laser-Doppler probe was placed to measure cortical cerebral blood flow.

**Figure 1:**
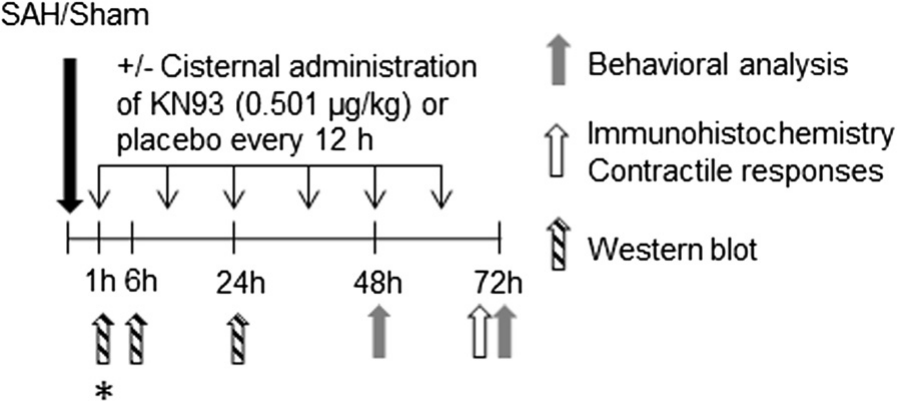
Experimental design. *Animals terminated at 1 h for evaluation of phosphorylated CaMKII with Western blot were treated with KN93 or placebo 45 min prior to and immediately after SAH.

A 27G blunt cannula with a side hole facing right was placed stereotactically 6.5 mm anterior to the bregma in the midline at an angle of 30° to the vertical plane placing the tip of the needle just in front of the chiasma opticum. The rat was placed on a heating pad connected with a rectal probe to keep the core temperature at 37.5 ± 0.5°C. After 30 minutes of equilibration, during which the level of anesthesia was adjusted to obtain a mean arterial blood pressure of 80 to100 mmHg, 250 μL of blood was withdrawn from the tail catheter and injected manually into the pre-chiasmatic cistern at a pressure equal to the mean arterial blood pressure. Subsequently, rats were maintained under anesthesia for another 60 minutes in order to allow them to recover. The ICP catheter was cut and closed with a removable plug (later to be used for administration of treatment) 2 cm from the tip, the tail catheter, the needle, and the laser-Doppler probe was removed and incisions closed. The rat was revitalized and extubated. At the end of the surgery and every 24 h until termination, the rat received a subcutaneous injection of Carprofen (4 mg/kg, Pfizer, Denmark) and 15 mL of isotonic saline for hydration. Sham-operated rats went through the same procedure, with the exception that no blood was injected intracisternally. In total, we included 70 animals in this study. The detailed number of animals is presented in the Results section. The experimental design is presented in Figure 1.

### KN93 and vehicle treatment groups

Rats in placebo and KN93 treatment groups went through the same surgical procedure as the above described SAH animals. Animals in the KN93 group were treated with 50 μL of a 10^−5^ M solution of KN93 (Cayman Chemicals, USA), yielding a final dose of 0.501 μg/kg body weight for a 330 g rat, diluted in isotonic saline +0.01% DMSO every 12 h with the first dose given 1 h post-SAH. The KN93 concentration was chosen based on earlier *in vitro* studies [19]. KN93 was delivered intracisternally through the ICP catheter with the tip placed in the cisterna magna. Animals in the placebo group were treated at the same time points with a corresponding volume of isotonic saline +0.01% DMSO via the same administration route.

For investigations of the CaMKII phosphorylation in cerebral arteries at 1 h after SAH, rats were treated with KN93 (dose as above) or placebo (as above) 45 minutes before induction of SAH and again immediately after induction of SAH. For investigations of CaMKII phosphorylation in cerebral arteries at 6 and 24 h after SAH, rats were treated with KN93 (dose as above) or placebo (as above) at 1 h post-SAH and repeated at 12 h post-SAH (for rats terminated at 24 h). An overview of the treatment regimen and experimental groups are presented in Figure 1.

### Rotating pole test

Gross sensorimotor function (integration and coordination of movements as well as balance) was evaluated by the ability of the rats to traverse a rotating pole on days 2 and 3 after SAH [23]. In one end of the pole (which was 45 mm in diameter and 150 cm in length) a cage with an entrance hole facing the pole was placed and the floor of the cage was covered with bedding material from the home cage of the rat being tested, thus serving as a positive reinforcement for the rat to traverse the pole when placed at the end opposite to the cage. The performance of the rats was scored according to the following definitions: score 1) the animal is unable to balance on the pole and falls off immediately; score 2) the animal balances on the pole but has severe difficulties crossing the pole and moves less than 30 cm; score 3) the animal embraces the pole with the paws and does not reach the end of the pole, but manages to move more than 30 cm; score 4) the animal traverses the pole, but embraces the pole with the paws and/or jumps with the hind legs; score 5) the animal traverses the pole with normal posture but with more than 3 to 4 foot slips; score 6) the animal traverses the pole perfectly with less than 3 to 4 foot slips. Each animal was tested on 0, 3, and 10 rotations per minute (rpm).

### Harvest of cerebral arteries

At 1, 6, or 24 h (for Western blot analysis of CaMKII phosphorylation) or at 72 h (for contractility measurements and immunohistochemistry of CaMKII and DREAM expression) after SAH or sham operation, rats were decapitated. The brains were quickly removed and chilled in cold bicarbonate buffer solution. The middle cerebral artery (MCA) and the basilar artery (BA) were carefully dissected from the brain.

### Western blot

The BA and both MCAs were isolated, cleared of blood and connective tissue, and snap frozen as one pooled sample of all three vessel segments from each rat. Each sample of frozen arteries were lysed in 70 μL boiling LDS sample buffer (Expedeon, Cambridgeshire, UK) containing 50 mM DTT, sonicated for 2 min and cleared by centrifugation at 15,000 at 4°C for 30 minutes. Then, 20 μL of each sample was separated by 4 to 20% SDS-PAGE (RunBlue, Expedeon, San Diego, CA, USA) and transferred to a PVDF membrane. Membranes were blocked in PBS with 0.1% Tween-20 and 5% bovine serum albumin (BSA) (Sigma-Aldrich, St Louis, MO, USA) for 1 h at room temperature and then incubated overnight at 4°C in blocking buffer with the primary antibody rabbit anti-rat phospho-CaMKII (Thr286; Cell Signaling Technology, Beverly, MA, USA) diluted 1:1,000, followed by incubation with ECL™ horseradish peroxidase-conjugated donkey anti-rabbit IgG antibody (GE Healthcare, Uppsala, Sweden) for 1 h at room temperature. Labelled proteins were developed using Lumigen TMA-6 chemiluminescence solutions (GE Healthcare). Subsequently, the membranes were incubated in stripping buffer, extensively washed in PBS +0.1% Tween-20, and then re-probed with the primary antibody mouse anti-rat CaMKII (Abcam, Cambridge, UK) diluted 1:200, at 4°C overnight in blocking buffer followed by incubation with horseradish peroxidase-conjugated goat anti-mouse IgG antibody (Pierce/Thermo Scientific, Rockford, IL, USA) 1:5,000 for 1 h at room temperature and development of labelled proteins. Labelling chemiluminescence intensities were quantified using the software Image Gauge V4.0 (Fujifilm, Tokyo, Japan). The quantitative data have been presented before (see below). Finally, as a loading control, membranes were washed and re-probed with mouse anti-actin antibodies followed by anti-mouse secondary antibodies as described above.

### *In vitro* pharmacology

For measurements of contractile responses of cerebral arteries, a wire myograph (Danish Myograph Technology A/S, Aarhus, Denmark) was used to record the isometric tension in segments of isolated cerebral arteries [24,25]. Briefly, 1 mm long vessel segments were mounted on two 40 μm diameter stainless steel wires in a set-up previously described [4]. The segments were immersed in a temperature-controlled buffer solution (37°C) of the following composition (mmol/L): NaCl 119, NaHCO_3_ 15, KCl 4.6, MgCl_2_ 1.2, NaH_2_PO_4_ 1.2, CaCl_2_ 1.5, and glucose 5.5. The buffer was continuously aerated with 5% CO_2_ resulting in a pH of 7.4. The vessel segments were stretched to an initial pretension of 1 to 2 mN/mm and allowed to equilibrate at this tension for 45 min. The vessels were then exposed to a solution of 63.5 mM K^+^ obtained by partial substitution of NaCl for KCl in the above described isotonic solution. The K^+^-evoked contractile responses were used as reference values for normalization of agonist-induced responses and to evaluate the contractile capacity of the vessels [24]. Only BAs with K^+^-induced responses over 2 mN and MCAs with K^+^-induced responses over 0.7 mN were included in the study. The presence of functional endothelium in the vessel segments was assessed by means of pre-contraction with 5-HT followed by relaxation with carbachol as previously described [9]. Concentration-response curves were obtained by cumulative application of 5-carboxamidotryptamine (5-CT) (Sigma-Aldrich) in the concentration range 10^−14^ to 3 × 10^−7^ M and ET-1 (AnaSpec, San Jose, CA, USA) in the concentration range 10^−14^ to 3 × 10^−7^ M.

### Immunohistochemistry

Vessel segments were embedded in Tissue TEK (Gibco, Invitrogen A/S, Taastrup, Denmark), frozen at −80°C, and cryosectioned (10 μm, Cryo-star HM 560 M Thermo Scientific, Microm, Germany). The sections were fixed for 10 min in −20°C acetone and rehydrated by 3 × 5 min phosphate buffered saline (PBS, pH 7.2) containing 0.25% Triton X-100 (PBST). The sections were incubated overnight at 4°C with primary antibodies, anti-DREAM (1:250, FL-214, sc-9142 Santa Cruz Biotechnology, Santa Cruz, CA, USA) and anti-CaMKII (1:100, ab-52476, Abcam) diluted in PBST containing 1% BSA. After incubation, sections were washed in PBST for 2 × 15 min and incubated for 1 h at room temperature with FITC-conjugated secondary antibody (1:100 Cayman Chemicals, Ann Arbor, MI, USA) diluted in PBST containing 1% BSA. The sections were then washed in PBST for 2 × 15 min and mounted with anti-fading mounting medium (DAPI-containing Vectashield; Vector Laboratories Inc., Burlingame, CA, USA). The same experimental procedure was used for negative controls, where primary antibodies were omitted. This resulted in no immunoreactivity except for auto-fluorescence in the internal elastic lamina. Immunoreactivity was visualized at the appropriate wavelength with an epifluorescence microscope (Nikon 80i; Tokyo, Japan) and photographed with an attached Nikon DS-2Mv camera. The fluorescence intensity was measured with the Image J software (http://rsb.info.nih.gov/ij/) in a blinded fashion by a second unbiased person. Each treatment group contained 5 to 7 rats, and 4 to 6 sections were evaluated for each rat. The fluorescence intensity was measured in four standardized areas (two, four, eight, and ten o’clock) in each section. The mean intensity per measured area was used as the fluorescence measurement. Results are shown as mean ± standard error of the mean and n refers to the number of vessels in each group for each antibody.

### Statistics

Data are presented as the mean ± SEM. Statistical analyses were performed using one-way ANOVA for the immunohistochemistry quantifications, two-way ANOVA with agonist concentration and experimental group as the two independent variables for the contractility curves, and non-parametric Kruskal-Wallis test for the rotating pole test. *P* <0.05 was considered significant.

## Results

### Subarachnoid hemorrhage (SAH) model

In all operated rats, mean arterial blood pressure, partial pCO_2_, partial pO_2_, pH, and body temperature were within normal physiological ranges during the operation. There was no statistical difference in physiological parameters among the groups. As a result of injecting blood, the cortical blood flow dropped over the right hemisphere to 10.3 ± 13% of resting flow and the ICP increased from 4.2 ± 5 mmHg to 128 ± 41 mmHg. The blood flow and ICP returned to basal values within 1 h after injection (for typical responses see [26]). There was no difference in ICP rise and cortical blood flow drop between treatment groups. The treatment with KN93 was well tolerated by the animals throughout the 72 h post-SAH period and did not induce any acute changes in the above parameters. There were no significant differences in the mortality rates between treatment groups.

### CaMKII phosphorylation in cerebral arteries

**Figure 2:**
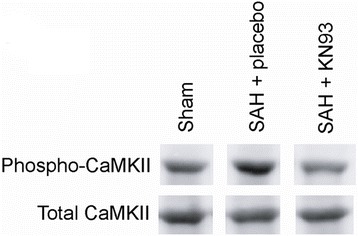
**Western blot of early CaMKII phosphorylation in cerebral arteries after SAH.** Representative blots of phosphorylated CaMKII and total CaMKII levels in cerebral arteries from rats terminated 1 h after SAH (given placebo or KN93) or sham-operation. KN93 or placebo were given 45 min prior to and immediately after SAH. Number of animals in each group, n = 4.

To assess whether CaMKII is activated in cerebral arteries after SAH, and to investigate the time-course of this activation, we performed Western blots of cerebral arteries from rats terminated at 1, 6, and 24 h after SAH or sham-operation with antibodies against CaMKII phosphorylated at Thr286 (indicating activation of CaMKII). Moreover, to test the effect of the CaMKII inhibitor KN93 on SAH-induced CaMKII activation (pCaMKII/total CaMKII; % of sham), we included a group of SAH rats treated with KN93 at each time point. As shown in Figure 2, a clear activation of CaMKII in cerebral arteries at 1 h post-SAH was observed (180 ± 29%; n = 4) and this SAH-induced CaMKII activation was prevented by treatment with KN93 45 min prior to and immediately after SAH (49 ± 11%; *P* <0.05). However, the SAH-induced CaMKII activation declined to sham-operated levels at 6 and 24 h post-SAH (at these time points there were no differences between the groups, data not shown) [27]. Thus, CaMKII activation appears to be an early, transient event in cerebral arteries after SAH. In support, elevation of phosphorylated CaMKII at 0.5 to 1 h after SAH induction was recently verified in a phosphoproteomic study [27]. We next continued to examine and understand the role of early CaMKII activation in arterial phenotypic changes and functional outcome after SAH.

### Protein levels of CaMKII and DREAM in cerebral arteries

**Figure 3:**
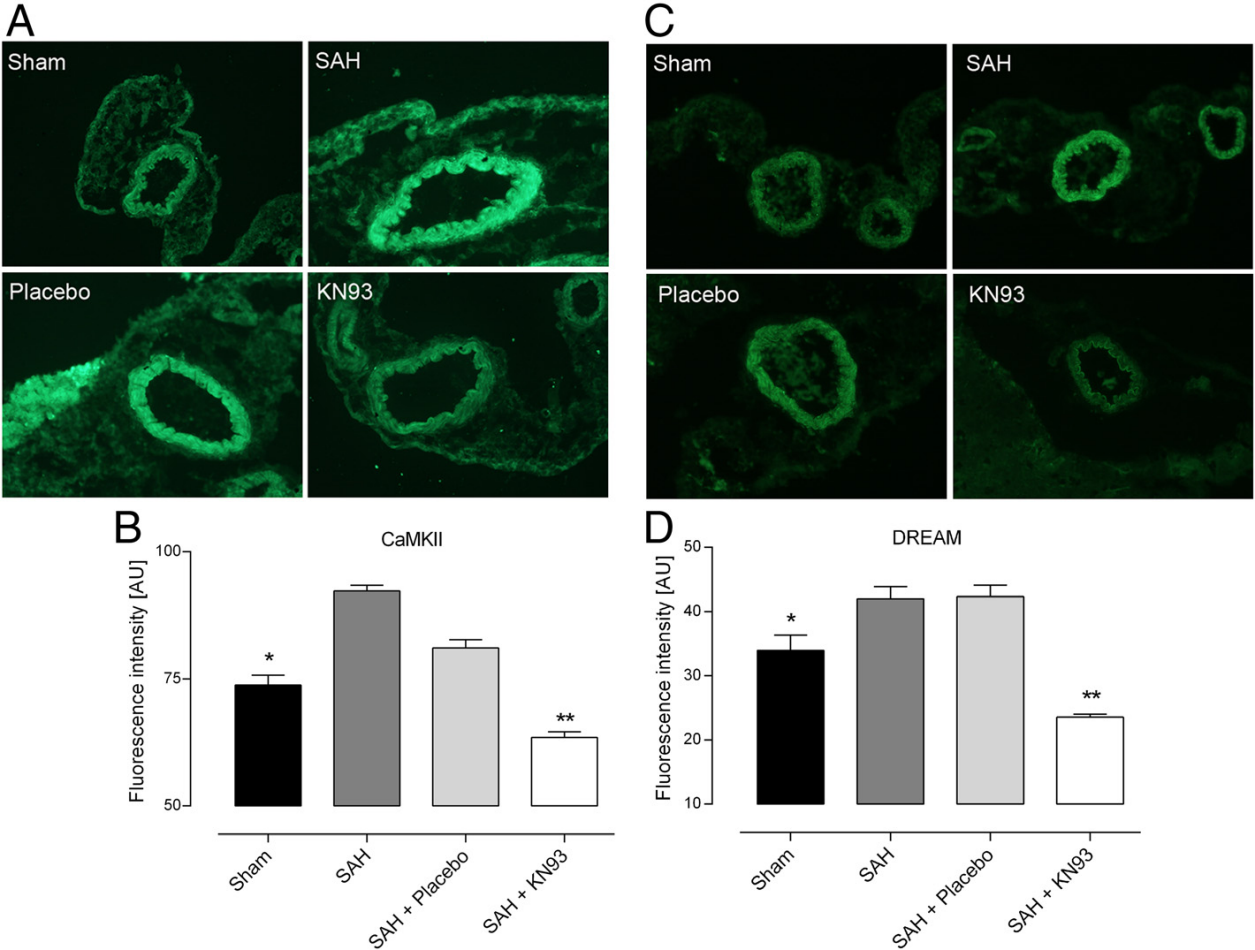
**Immunohistochemistry evaluation of the CaMKII and DREAM expression in cerebral arteries.** **(A,** **B)** Markedly increased CaMKII protein expression at 72 h post-SAH. KN93 decreased the CaMKII expression significantly. **(C,** **D)** Increased DREAM protein expression in SAH and SAH + Placebo groups. Administration of KN93 decreased the DREAM immunoreactivity significantly. The protein expression of CaMKII and DREAM in the different groups was compared to SAH as control. No staining, except for autofluorescence in the internal elastic lamina, was observed in the negative controls were primary antibodies were omitted (data not shown). *P <0.05 and **P <0.01 vs. SAH. Values are presented as mean ± SEM. Number of animals in each group, n = 5–7.

Immunohistochemistry was used to investigate whether SAH affects CaMKII expression in cerebral arteries. As shown in Figure 3A,B, CaMKII protein expression increased in cerebral vascular smooth muscle cells after SAH compared to sham (*P* <0.05). Interestingly, KN93 significantly decreased the CaMKII immunoreactivity compared to SAH (*P* <0.01, Figure 3A,B). We have previously shown that organ culture of cerebral arteries induces CaMKII activation [10,19], which in turn induces increased expression of the DREAM transcription repressor. Here, we investigated if SAH-induced CaMKII activation also affects downstream DREAM protein expression.

A significant increase in the DREAM immunoreactivity was observed after SAH compared to sham (*P* <0.05, Figure 3C,D). Treatment with KN93 prevented the increase in DREAM protein expression compared to the untreated SAH group or SAH treated with placebo (*P* <0.01, Figure 3C,D).

### Contractile responses of cerebral arteries

**Figure 4:**
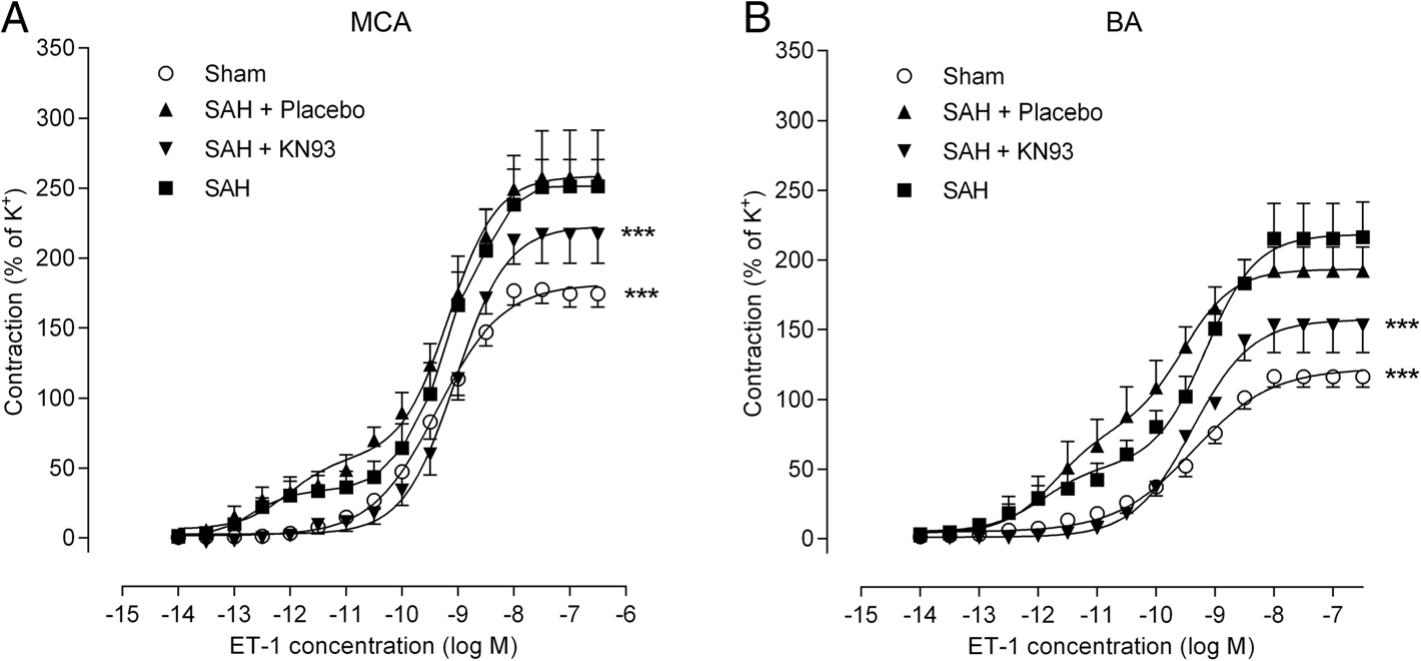
**Cerebrovascular contractile responses to ET-1 at 72 h post-SAH.** Concentration-response curves were elicited by the cumulative application of ET-1 to **(A)** MCA and **(B)** basilar arteries. In both arteries, the first phase of the concentration-response curve represents ET-1-induced contraction via ET_B_ receptors and the second phase contraction mediated by ET_A_ receptors. Previous studies have characterized these responses in detail [4,9,28]. Values are expressed as percentage of the K^+^-induced contraction and are presented as mean ± SEM. ***P <0.001. Number of animals in each group, n = 6–8.

Potassium (63.5 mM) induced a potent vasoconstriction in all vessels and was used as an internal control set to 100%. There was no difference in the potassium-induced responses between the groups studied. Contractile responses to cumulative administration of ET-1 (10^−14^ to 10^−7^ M) are shown in Figure 4. In cerebral arteries from sham-operated rats, ET-1 induced sigmoidal concentration-response curves in both MCA and BA. SAH (untreated or placebo) resulted in leftward shifts, and a change from sigmoidal to biphasic concentration-response curves. This is in accordance with our earlier studies, which demonstrated that the first phase of the ET-1 concentration-response curves represents contraction mediated by contractile ET_B_ receptors upregulated in the smooth muscle cells after SAH, whereas the second phase of the curve represents vasoconstriction mediated by ET_A_ receptors [9]. The change into a biphasic curve together with the increased E_max_ values after SAH suggests upregulation of both ET_A_ and ET_B_ receptors after SAH. The detailed pharmacological characterizations have been published before [4,9,28]. Treatment with KN93 totally abolished the elevation of the first phase but not the second phase, thus yielding ET-1 concentration-response curves similar to the vascular contractile response seen in arteries from sham operated animals. This indicates that KN93 prevents the expression of ET_B_ receptors after SAH.

**Figure 5:**
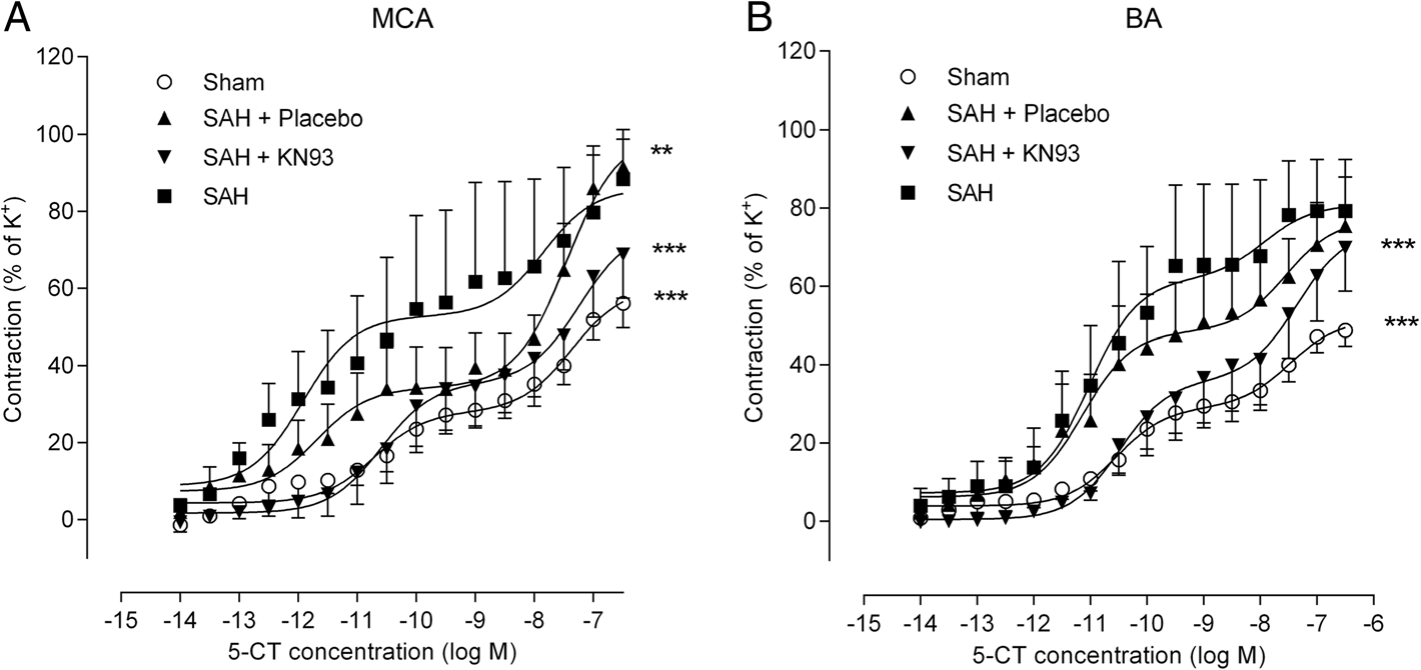
**Cerebrovascular contractile responses to 5-CT at 72 h post-SAH.** Concentration-response curves were elicited by the cumulative application of 5-CT to **(A)** MCA and **(B)** basilar arteries. The 5-CT induced contractile responses show two phases; the first part represents elevation in the 5-HT_1B_ receptor expression and the second part shows unaltered 5-HT_2A_ receptor function in MCA and BA after SAH. The detailed pharmacological characterizations have been previously published [4,9,28,29]. Values are expressed as percentage of the K^+^-induced contraction and are presented as mean ± SEM. **P <0.01 and ***P <0.001. Number of animals in each group, n = 6–8.

Contractile responses to cumulative administration of 5-CT (10^−14^ to 3 × 10^−7^ M) are shown in Figure 5. 5-CT yielded biphasic concentration-response curves in both MCA and BA, mediated by the presence of 5-HT_1B_ (first phase of the concentration-response curve) and 5-HT_2A_ (second phase) receptors as previously characterized in detail [29]. Contraction induced by 5-CT in cerebral arteries from SAH rats (untreated or placebo) showed leftward shifts and increased E_max_ values compared to sham, indicating upregulation of cerebrovascular 5-HT receptors after SAH. Treatment with KN93 abolished the elevation of the first phase but not the second phase, yielding 5-CT concentration-response curves as seen in arteries from sham-operated animals. This indicates that KN93 prevents the enhanced expression of 5-HT_1B_ receptors after SAH.

### Sensorimotor function

**Figure 6:**
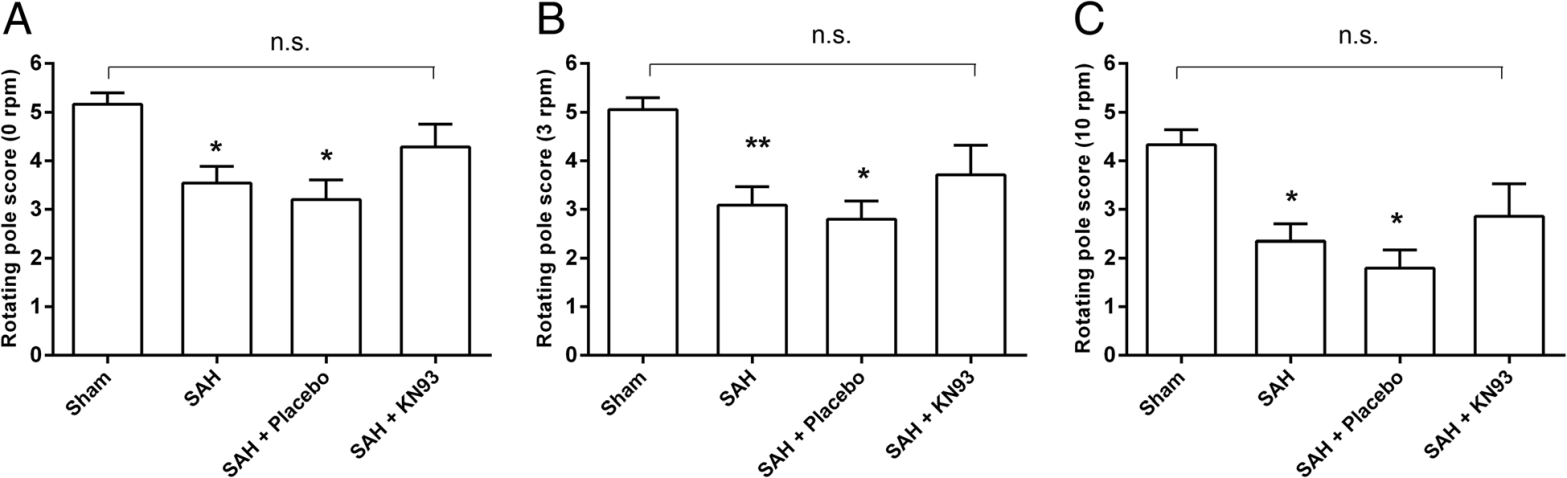
**Sensorimotor function 2 days after SAH.** Animals were tested on the rotating pole at **(A)** 0 rotations per minute (rpm), **(B)** 3 rpm, and **(C)** 10 rpm. *P <0.05 and **P <0.01 compared to sham. Animals were given a maximum score of 6 if they could traverse the pole perfectly with less than 3 to 4 foot slips. Values are presented as mean ± SEM. Number of animals in each group, n = 6–13.

The conclusion from the clazosentan treatment trial program was that CVS was not the key endpoint but the outcome of SAH, indicating the importance of DCI as the best endpoint in evaluation of trials. Therefore, we used the integration and coordination of movements as evaluated by the rotating pole test [23]. When tested 2 days after SAH or sham-operation, sham rats obtained an average score of 5.2 ± 0.2 in the no rotation test, whereas animals in SAH and SAH with placebo groups obtained significantly lower scores (3.5 ± 0.3 and 3.2 ± 0.4, respectively, *P* <0.01) in the no rotation test (Figure 6A). KN93 treatment prevented the worsened sensorimotor function after SAH and treated rats had an average score of 4.3 ± 0.5, which was not significantly different from the sham group. Performing the test with rotation at 3 rpm and 10 rpm decreased the performance of the rats in all groups (Figure 6B,C). However, untreated and placebo-treated SAH rats obtained lower scores than sham-operated rats and KN93-treated SAH rats. These data suggest improved gross sensorimotor function after treatment with the CaMKII inhibitor in SAH. Treatment of sham rats with KN93 did not show modified responses as compared to sham rats (data not shown). At 3 days after SAH or sham-operation, the results of the rotating pole test were similar to results obtained at day 2 but less marked between groups (data not shown).

## Discussion

The etiology of CVS and DCI after SAH remains an unresolved issue. Involvement of mitogen activated protein kinases (MAPK) and, in particular, the MEK/ERK1/2 pathway has been demonstrated, and early inhibition of these protein kinases may significantly alleviate DCI and improve outcome after experimental SAH in rats [4,28]. This was strongly supported by a study of signal-transduction mechanisms activated after SAH in a phosphoproteomic study with quantitative mass spectrometry [27]. The CaMKII and MEK/ERK1/2 pathways were activated at 0.5 and 1 h after the SAH and seemed to correlate in a time-dependent manner. Recently, we reported evidence for a cross-talk between these two kinase systems using an organ culture method [10]. Because of this work, designed to understand the details of intracellular mediators activated after SAH, we asked the current question of whether CaMKII has a role in induction of vasoconstrictor receptor upregulation and if its inhibition may improve outcome after SAH. Organ culture has been used as a model that has allowed for a detailed molecular mechanistic analysis aimed at evaluating the role of different kinases, including MAPK and CaMKII, on the transcriptional upregulation of endothelin and 5-HT receptors [30]. These studies have shown that CaMKII is activated in rat cerebral arteries [19] after organ culture and *in vivo* after different types of stroke [27]. Here, we report that phosphorylated CaMKII is elevated early after induction of SAH, at 1 h, but transiently. In addition, the inhibition of CaMKII with KN93 has previously been demonstrated to decrease the mRNA level of endothelin ET_A_ and ET_B_ receptors, and to reduce ET-1 and sarafotoxin 6c (ET_B_ receptor-specific agonist)-induced contractions [19].

The present study is thus the first to demonstrate the *in vivo* effect of the CaMKII inhibitor KN93 in a well-characterized rodent SAH model [28]. The *in vivo* results show that KN93 administration attenuated the SAH-induced elevation of contractions induced by ET-1 (mediated by ET_A_ and ET_B_ receptors) in BA and MCA arteries. In addition, KN93 treatment abolished the first phase in the biphasic concentration-response curve induced by 5-CT, indicating prevention of 5-HT_1B_ receptor upregulation after SAH. We observed that KN93 successfully attenuated contractions induced by ET-1 and 5-CT. In support, it has been shown that the circulatory levels of 5-HT and ET-1 increase after SAH [6]. Thus, the inhibitory effect of KN93 on contractions induced by 5-CT and ET-1 suggests an important role of CaMKII in mediating the elevated vascular contractions after SAH.

It has also been shown that [Ca^2+^]_i_ and CaMKII cooperatively regulate the subcellular localization into the nucleus and thereby promotes downstream DREAM-induced transcriptional repression [31]. The present results show, for the first time, that SAH induced increased arterial DREAM expression, and this expression was significantly attenuated following KN93 treatment. Taken together, the results support the involvement of CaMKII in downstream regulation of DREAM at the transcriptional level after experimental SAH.

Evaluation of the gross sensorimotor function after SAH showed that KN93 had an effect on the neurological outcome after SAH. Treatment with KN93 at 1 h and every 12 h post-SAH prevented worsened neurological function at 2 days (seen in SAH and SAH + placebo animals) and similar test scores were achieved as in sham-operated rats. Although KN93-treated rats tended to have higher scores than SAH and SAH + placebo, this difference was not significantly different. The dose of KN93 (0.501 μg/kg) was based on a previous *in vitro* study using the organ culture method. This concentration is low compared to another study that administered a different CaMKII inhibitor at 1 mg/kg intravenously for the treatment of ischemic stroke in rats [20]. It may be interesting to investigate higher concentrations and other available CaMKII inhibitors to evaluate if they may have stronger effects on the neurological outcome after SAH.

## Conclusions

The present study has revealed the involvement of CaMKII in the events occurring after SAH. Increased vasoconstriction to ET-1 and 5-CT was observed in cerebral arteries, as well as elevation of vascular smooth muscle CaMKII and DREAM protein expression. Treatment with the CaMKII inhibitor KN93 significantly attenuated the increase in contractile responses and in CaMKII and DREAM protein levels and prevented sensorimotor deficits after SAH.

## Abbreviations

BA: Basilar artery
BSA: Bovine serum albumin
CaMK: Calcium-calmodulin dependent protein kinase
CVS: Cerebral vasospasm
DREAM: Downstream regulatory element antagonist modulator
DCI: Delayed cerebral ischemia
ET_A_: Endothelin A
ET_B_: Endothelin B
ET-1: Endothelin-1
ICP: Intracranial pressure
MAPK: Mitogen activated protein kinase
MCA: Middle cerebral artery
pCO_2_: Partial pressure of carbon
rpm: Rotations per minute
SAH: Subarachnoid hemorrhage
5-CT: 5-Hydroxycarboxamide
5-HT: 5-Hydroytryptamine
